# Intermetallic compounds in catalysis – a versatile class of materials meets interesting challenges

**DOI:** 10.1080/14686996.2020.1758544

**Published:** 2020-06-15

**Authors:** Marc Armbrüster

**Affiliations:** Faculty of Natural Sciences, Institute of Chemistry, Materials for Innovative Energy Concepts, Chemnitz University of Technology, Chemnitz, Germany

**Keywords:** Intermetallic compounds, heterogeneous catalysis, gas-phase catalysis, electrocatalysis, 106 Metallic materials, 102 Porous / Nanoporous / Nanostructured materials, 205 Catalyst / Photocatalyst / Photosynthesis, 206 Energy conversion / transport / storage / recovery, 212 Surface and interfaces, 301 Chemical syntheses / processing, 302 Crystallization / Heat treatment / Crystal growth, 306 Thin film / Coatings, 500 Characterization

## Abstract

The large and vivid field of intermetallic compounds in catalysis is reviewed to identify necessities, strategies and new developments making use of the advantageous catalytic properties of intermetallic compounds. Since recent reviews summarizing contributions in heterogeneous catalysis as well as electrocatalysis are available, this contribution is not aiming at a comprehensive literature review.

To introduce the field, first the interesting nature of intermetallic compounds is elaborated – including possibilities as well as requirements to address catalytic questions. Subsequently, this review focuses on exciting developments and example success stories of intermetallic compounds in catalysis. Since many of these are based on recent advances in synthesis, a short overview of synthesis and characterisation is included. Thus, this contribution aims to be an introduction to the newcomer as well as being helpful to the experienced researcher by summarising the different approaches. Selected examples from literature are chosen to illustrate the versatility of intermetallic compounds in heterogeneous catalysis where the emphasis is on developments since the last comprehensive review in the field.

## Graphical abstract

**Figure:**
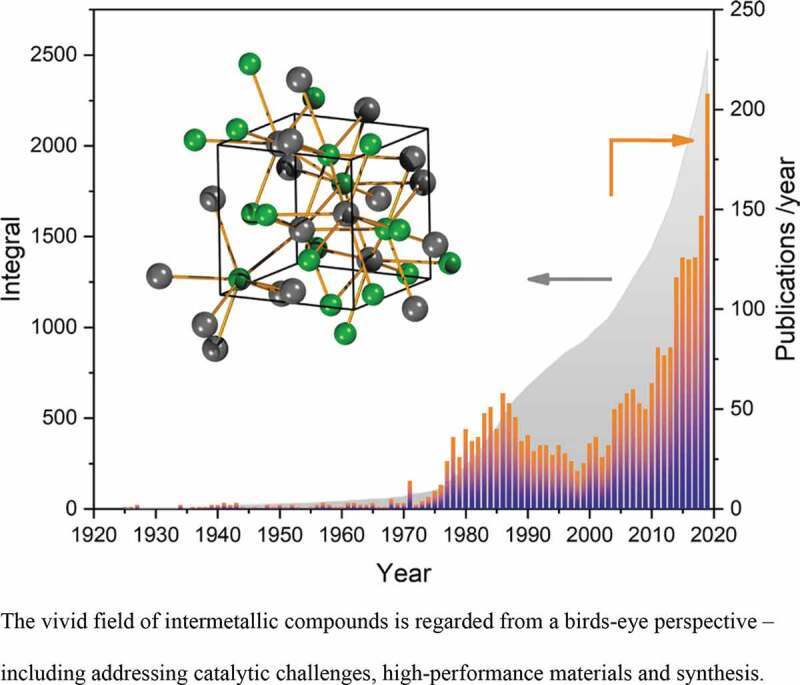

The vivid field of intermetallic compounds is regarded from a birds-eye perspective – including addressing catalytic challenges, high-performance materials and synthesis.

## 1. The nature of intermetallic compounds

Intermetallic compounds have fascinated [1–5] scientists from different fields for many decades. Physicists are intrigued by the broad variety of electronic properties, e.g. superconductivity [6] or magnetism [7], crystallographers are encountering very complex structures with up to 23,704 atoms in the unit cell [8] or even quasicrystalline phases [9]. Linus Pauling addressed intermetallic compounds in his well-recognised book *The Nature of the Chemical Bond and the Structure of Molecules and Crystals* [10] and pointed out that this branch of structural chemistry needed further development [11]. Since then, numerous scientists tried to understand the reasons behind the complex crystal structures and developed ‘rules’ for some of them (Hume-Rothery phases [12–16], Laves phases [17–19] and the Zintl-Klemm-Busmann concept [20–27]). While these hold in many, but only special cases, there is so far no comprehensive understanding or at least a theory to understand or even predict structures. Investigations in the past revealed that the chemical bonding in the intermetallic compounds is unique in most of the compounds and even isostructural compounds need not be based on the same chemical bonding pattern [28]. Nevertheless, the chemical bonding, i.e. the bonding interaction between atoms, determines the electronic structure and thus the physicochemical properties to a large extent.

**Figure 1.**
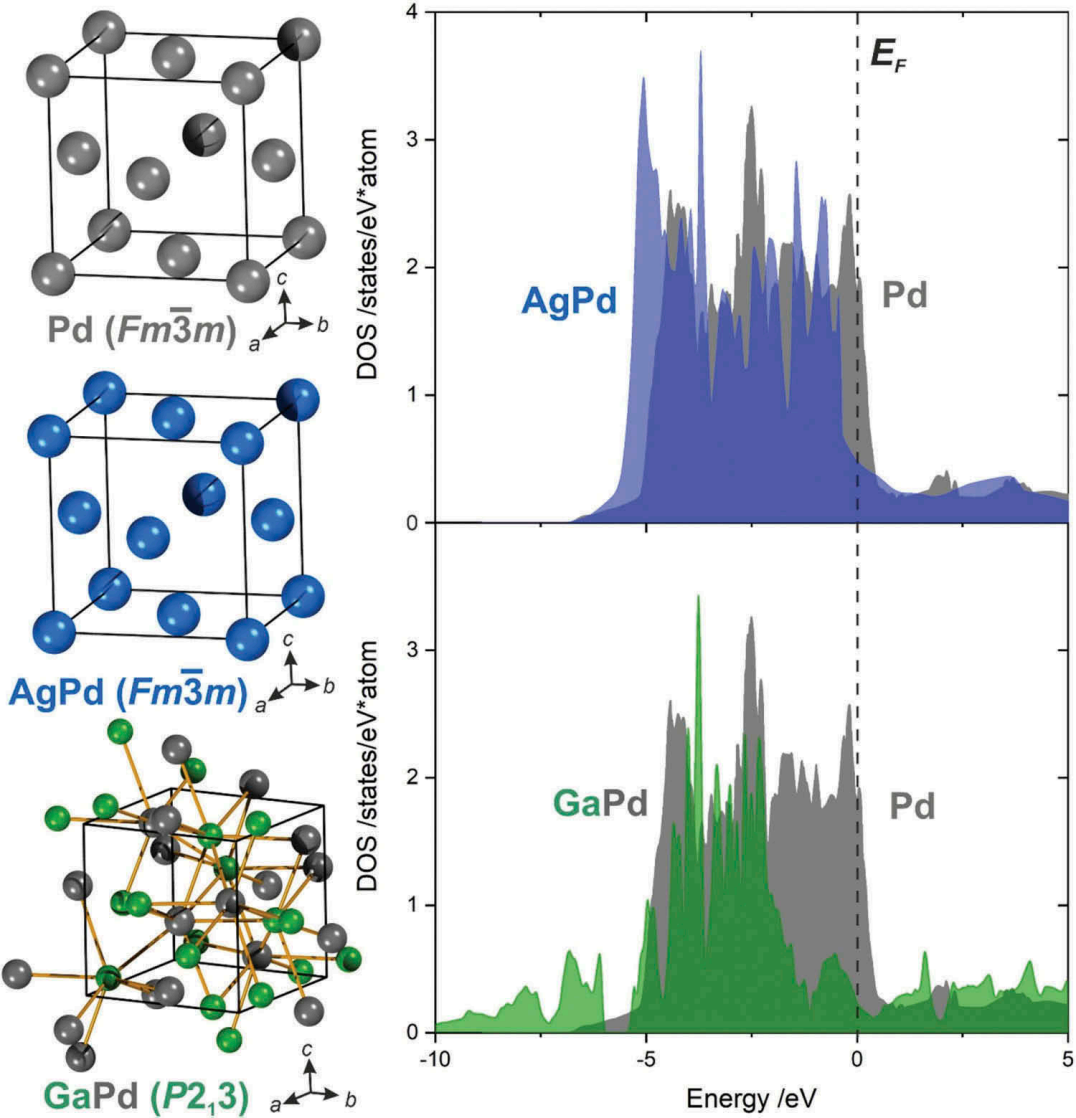
Comparison of the unit cells of elemental palladium, the substitutional alloy AgPd and the well-ordered intermetallic compound GaPd (left). For the first two, the electronic structure mostly differs by the filling-degree, while the crystal structure of GaPd offers a very different scaffold for the electronic structure due to the altered arrangement of the atoms in space (right).

Characteristic for intermetallic compounds, i.e. compounds formed by at least two metals (usually boron, silicon, arsenic and tellurium are also considered as forming intermetallic compounds with the more typical metals), are their – at least partly – ordered crystal structures which are different from the ones of the constituting elements (Figure 1)[29]. The ‘at least partly ordered’ excludes substitutional alloys, since here all atoms are randomly occupying the crystallographic sites of the crystal structure of one of the constituting elements. The reason to distinguish between the two, i.e. intermetallic compounds and substitutional alloys, is that intermetallic compounds possess more complex crystal structures than the Cu- (ccp: cubic close packed), Mg- (hcp: hexagonal close packed) or W- (bcc: body centred cubic) type of crystal structure of the metallic elements. Thus, intermetallic compounds offer new ‘scaffolds’ for the electronic structure (Figure 1) and with it the potential to discover new properties. This especially holds for the chemical properties, e.g. catalytic properties, which form a rather young but very vivid field.

The special properties of intermetallic compounds are caused by their peculiar combination of electronic and crystal structure, leading to chemical potentials of the elements not easily obtained in other compounds. The chemical potential results to a large extent from the electron transfer between the constituting elements, which in turn depends on the electronegativity difference. Thus, by choosing elements with appropriate properties, the chemical potential of a (transition)metal can be adjusted within a broad range while keeping electrical conductivity. Palladium, a rather frequent player in heterogeneous catalysis, might serve as an example for the versatility. Palladium is well known in an oxidised or elemental form, e.g. formally as Pd^2+^O^2-^ or Pd^0^, respectively. The chemical potential can be adjusted between these two states (‘2+’ and ‘0’) by changing the bonding partner. Sulfur as bonding partner will lead to a positive charge on palladium; using fluorine the positive charge will be even higher due to the larger electronegativity difference. But how to create a negatively charged palladium, thus opening new chemistry? For this, a more electropositive bonding partner is needed, restricting the choice to metals, e.g. gallium, and resulting in intermetallic compound formation. In intermetallic Ga-Pd compounds (one of the first intermetallic compounds used to apply specific properties of intermetallic compounds in catalysis), experiments as well as quantum chemical calculations have shown that electrons are transferred from gallium to palladium[30]. The electron transfer is based upon the electronegativity difference and is a general phenomenon in intermetallic compounds[31]. Thus, the chemical potential of a metal *M* within an intermetallic compound can be adjusted between the tellurides (positively charged *M*) and the compounds with caesium (negatively charged *M*) upon intermetallic compound formation.

Thus, the class of intermetallic compounds allows testing structural influences as well as electronic influences on their physical and chemical properties. When addressing the first, compounds with appropriate structural motifs have to be chosen. For the second, either isostructural compounds with different elements can be used or one of the constituting elements is partly replaced by one with a different valence electron count – if the crystal structure is not changed, i.e. if an isostructural substitutional series exists.

Last but not least, intermetallic compounds can be synthesised as supported or unsupported materials, polycrystalline bulk or powder samples or cm-sized single crystals. This enables a wide variety of materials opening experimental investigations ranging from classical catalysis to surface science. This is further enhanced by combining experimental with quantum chemical methods for which the well-described surfaces of the single crystals are an excellent starting point. Intermetallic compounds are thus platform materials significantly narrowing the ‘materials gap’[32] and leading to deep understanding.

## 2. Catalysis and intermetallic compounds

**Figure 2.**
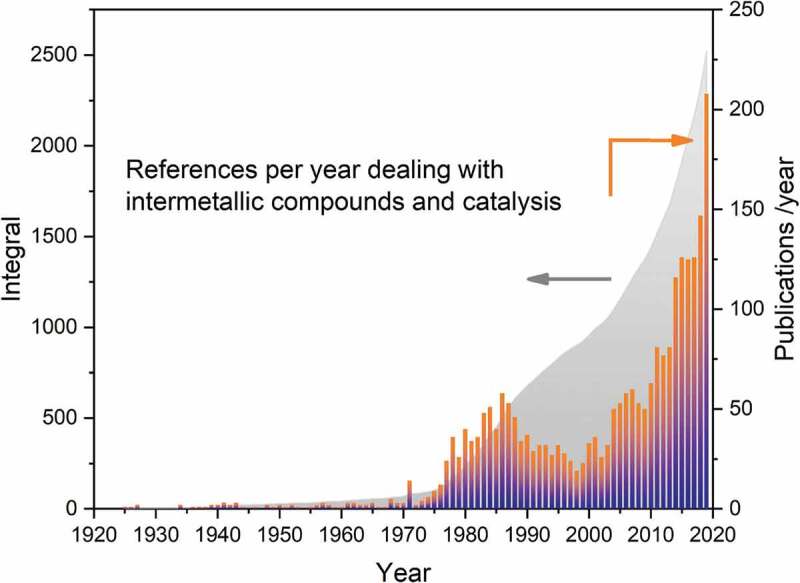
Number of relevant publications per year proving the presence of ordered intermetallic compounds and investigating catalytic properties known to the author.

While chemical properties comprise many facets, much effort has been brought forward to explore and investigate the catalytic properties of intermetallic compounds in various reactions. Contributions to the field are not always easy to find in literature databases since terms like ‘bimetallic catalyst’, ‘promoted/modified catalyst’ or ‘alloy’ (just to name a few) are used in publications instead of ‘intermetallic compound’. Thus, a search in the Web of Science combining the strings ‘intermetallic’ and ‘cataly*’ results in 1,475 hits out of which a significant number are dealing with ‘intermetallic alloys’ representing substitutional alloys, thus no intermetallic compounds. Analysis of the potential literature for proof of the characteristic ordered crystal structure reveals at least 2,500 publications in the field – starting as early as 1925 with the patents filed by Raney [33,34] (Figure 2). In 2019, the number of publications dealing with intermetallic compounds in catalysis was more than 200, with an increasing tendency to use the term ‘intermetallic compound’ in the correct way thus easing the search for references.

Intermetallic compounds have been applied in an unsupported and supported state to systematically investigate ligand and geometric effects as well as the influence of the support – in recent years especially in the semi-hydrogenation of acetylene [35,36], methanol synthesis [37] and methanol steam reforming [38]. Due to their electrical conductivity, intermetallic compounds are also applicable in the field of electrocatalysis, enhancing the combined experimental and quantum chemical approach summarised by Jaramillo et al. [39] Controlled leaching [40] and development of electrodes with higher stability [41] based on the Brewer-Engel bonding theory [42] are just two examples. By now, the community has increased enormously, especially of those that are testing supported materials. This is reflected in the large number of reviews that have been published in the field in the recent years, focusing on nanomaterials [43], electrocatalytic properties [4], gas-phase catalysis [1] – just to name the most recent ones – as well as the IMCAT (Intermetallic Compounds in Catalysis) conferences taking place since 2010.

What makes intermetallic compounds so interesting for the investigation of their catalytic properties? Heterogeneous catalysis is dominated by two material properties, i.e. the electronic structure (ligand effect) and the arrangement of the atoms (geometric effect). A priori, any material offering altered electronic or structural properties is of interest for heterogeneous catalysis. While intermetallic compounds fulfil these criteria, they offer three advantages compared to substitutional alloys: i) **stability**, caused by the chemical bonding [5], which can prevent segregation or decomposition in reactive atmosphere, ii) a **wide range of chemical potential** of the involved (transition)metals and iii) peculiar combinations of electronic and crystal **structure**. The stability allows studying their intrinsic properties in many environments, while the different available chemical potentials can be used to adjust the redox properties to the needs of the reaction in question. Systematic study of the geometric effects in reactions is possible by exploiting the large variety of available crystal structures. Finally, the use of isostructural compounds or isostructural substitutional series with different valence electron concentration allows addressing the electronic effect (ligand effect) on a reaction. Since only the filling degree of the electronic structure changes, while the crystal structure and thus the electronic scaffold is kept, the electronic effect can be studied with only minor influence of the geometric effect. Like the metallic elements and the substitutional alloys, intermetallic compounds possess electrical conductivity, allowing electrochemical studies – even though the conductivity is often an order of magnitude less, due to the covalent and ionic interactions (so-called ‘bad metals’[44]).

However, the full use of these advantages comes at a price. To be able to assign the observed catalytic properties to the compound in question, the state of the material must be clarified while the catalytic reaction is proceeding, i.e. under *operando* conditions. Nevertheless, even if *operando* methods are not available, the straightforward structural characterisation of the materials before and after catalysis, e.g. by powder X-ray diffraction, already gives valuable information whether changes to the material occurred during catalysis. The structural characterisation is of central importance since the properties of the crystal structure are the decisive characteristic for the presence of an intermetallic compound.

**Figure 3.**
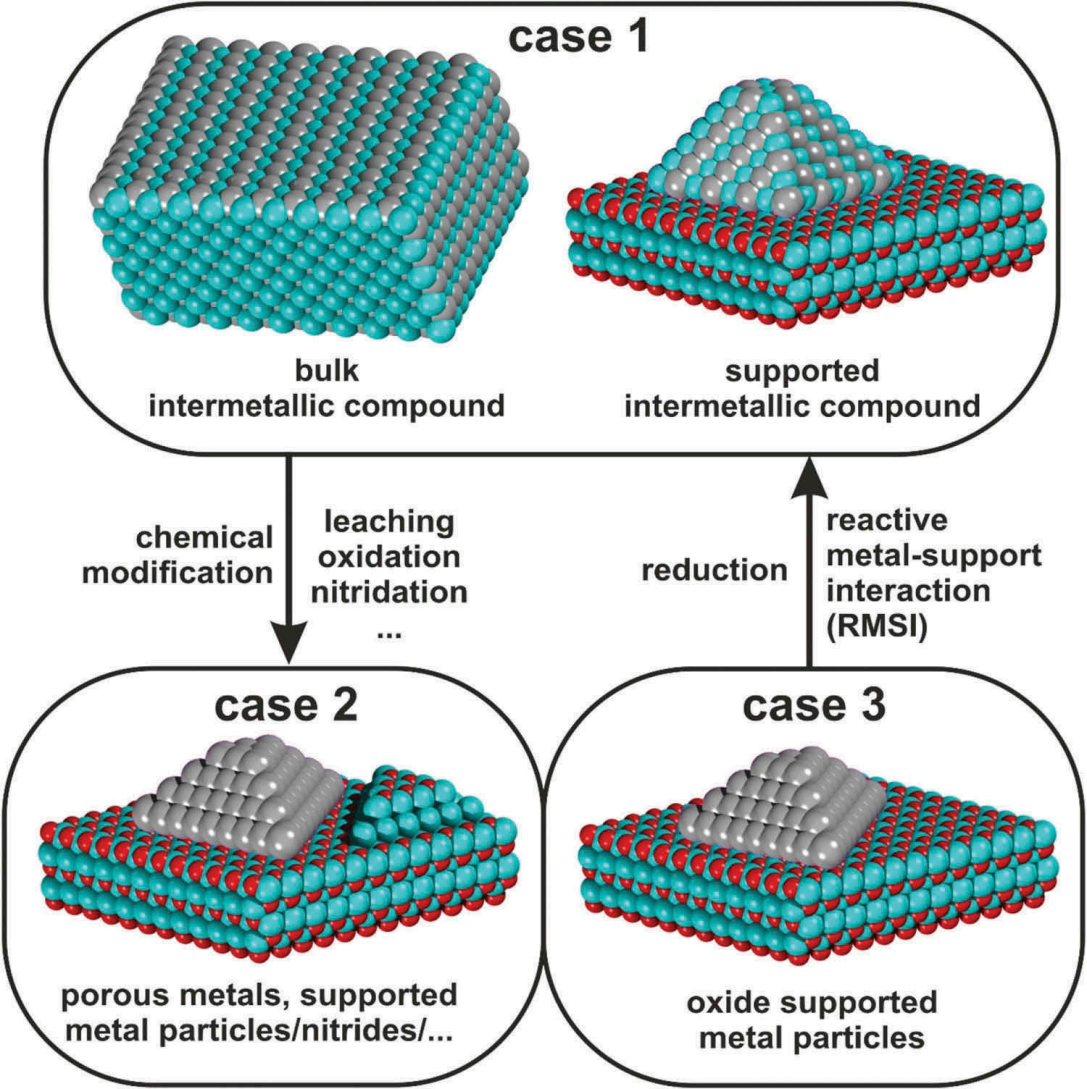
Three cases in which intermetallic compounds can be relevant for catalysis: as such (case 1), as precursors (case 2) or formation during catalysis by reactive metal-support interaction (RMSI) (case 3).

Intermetallic compounds can be relevant for catalysis in three ways (Figure 3). First, they can be used as such, either in an unsupported or supported state, and are stable under reaction conditions (case 1). In this case, the intermetallic compound is synthesised and structurally characterised at least before and after studying the catalytic properties. Exchanging the supporting material allows addressing the contribution of the support material to the catalytic properties. Intermetallic compounds can also be precursors and decomposed to the catalytically active species by (partial) oxidation or leaching (case 2). Raney catalysts are a prominent example where caustic leaching of Al-Ni or Ni-Si intermetallic compounds leads to nickel with high specific surface area, which is then applied as hydrogenation catalyst[45]. A trickier example is the formation of intermetallic hydrides under reaction conditions[46]. The hydride may only be stable under reaction conditions, so the structural characterisation before and after the reaction results in the detection of the intermetallic compound while actually the hydride is the catalytically active species. Rather often, the oxidation of intermetallic compounds by one of the reactants under reaction conditions has been described. An early example is the work of Wallace who explored the methanation of carbon monoxide using *M*Ni_5_ (*M* = La, Ce, Pr and Th) intermetallic compounds[47]. Since the rare-earth metals are strongly oxophilic and even in intermetallic compounds often easy to oxidise, the materials decompose under reaction conditions to elemental nickel supported on the corresponding rare-earth oxide. Decomposition is not restricted to hydride or oxide formation, the formation of intermetallic nitrides has also been reported[48]. Thus, in case 2 the observed catalytic properties can not be assigned to the intermetallic compound but are due to the reaction products (elements, hydrides, oxides, …). Last but not least, intermetallic compounds can be formed under reaction conditions (un)intentionally (moving from case 3 to case 1). The ingredients needed are metal particles, e.g. Pd, supported on an oxide, which can be (partially) reduced under reaction conditions, e.g. In_2_O_3_, and the corresponding reducing conditions, e.g. hydrogen-containing atmosphere (either as reactants or product of the catalytic reaction) and elevated temperature. If the temperature is sufficient, the hydrogen is activated by the metal and reduces the oxide near the metal particle. Subsequently, the resulting metal atoms diffuse inside the metal particle and form the phases corresponding to the binary phase diagram, i.e. substitutional alloys or intermetallic compounds. This effect is the so-called reactive metal-support interaction for which an extensive review is available[49].

Very recently, supported bi-metallic melts were introduced as catalytic materials by Wasserscheid et al. [50]. These supported catalytically active metal solutions (SCALMs) are liquid at reaction temperature and expose single atoms on the surface, thus preventing coking during dehydrogenation of alkanes. While intermetallic compounds can occur in these materials at ambient temperature, they are not present under reaction conditions and do not participate in catalysis. Thus, this interesting class of materials is not further considered in this review. The same holds for the recent development of testing high entropy alloys (HEA) in catalysis[51]. Single-phase HEA consist usually of five elements with close-to equiatomic ratios and represent substitutional alloys, i.e. random positioning of the different atoms on a close packed cubic or hexagonal lattice. Still, they offer the possibility for developments in heterogeneous catalysis. Providing an enormous number of different active sites, they allow for a screening – if the active site can subsequently be identified[52].

## 3. Addressing catalytic challenges

Efforts related to the use of intermetallic compounds in catalysis in the past years can be sorted in three categories. In the first group of contributions, intermetallic compounds are deployed to gain understanding of the needs of a reaction. The second category comprises the use of intermetallic compounds as high-performance materials and finally yet importantly, intermetallic compounds are applied as new catalytic materials for known and new reactions.

### 3.1. Deep understanding

As shown above, intermetallic compounds are especially useful to gain knowledge about the electronic and structural needs of a reaction. In addition, they can be applied as supported materials, thus enabling studies on synergistic effects between the intermetallic compound and the supporting material. While the benefit of such studies is enormous, their number is rather small. The reason is the effort which has to be brought forward for the targeted synthesis and thorough characterisation of the materials before and after reaction. In addition, such studies require the characterisation of the materials under *operando* conditions, i.e. while the catalytic reactions take place. *Operando* studies are the only way to identify and resolve possible material changes and to clarify which phases are present and relate them to the catalytic properties. Intermetallic compounds have been applied successfully to learn about the needs of several reactions and thus to enhance the knowledge as well as to create materials with superior catalytic properties. This comprises selective hydrogenation, methanol steam reforming, partial oxidation as well as electrocatalysis, thus covering a large range of redox-active atmospheres.

**Figure 4.**
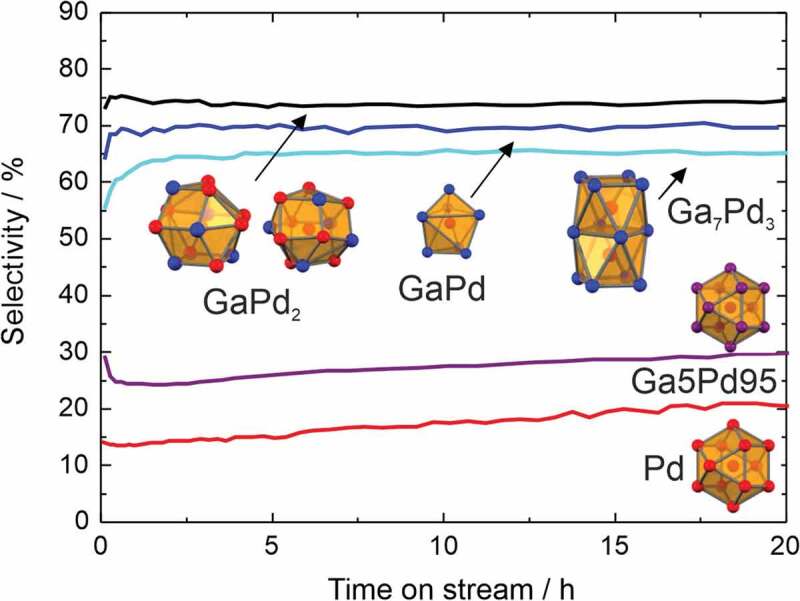
Coordination of palladium in the intermetallic compounds GaPd_2_, GaPd, and Ga_7_Pd_3_ (Pd: red, Ga: blue, pink: mixed) and selectivity to ethylene in the semi-hydrogenation of acetylene at 95% conversion (200°C, 0.5% C_2_H_2_, 5% H_2_, 50% C_2_H_4_ in helium).

Systematic studies of the selective semi-hydrogenation of acetylene (C_2_H_2_+ H_2_ → C_2_H_4_) on unsupported intermetallic compounds started back in 2006, correlating geometry and selectivity. Studies were conducted under simulated industrial front-end conditions (1:10:100 ratio of acetylene, hydrogen and ethylene) after steam cracking, thus mimicking relevant conditions for the cleaning of the ethylene stream. According to the active-site isolation concept, developed in the 1970s by Sachtler and others [53], small and spatially separated active sites should result in high selectivity to ethylene without hydrogenation of the excess ethylene. Intermetallic Ga-Pd compounds fulfilling the active-site isolation concept (Figure 4) were selected due to the covalent bonding interactions [54] resulting in high stability under reaction conditions as proven by a large variety of different *operando* methods [55–58].

**Figure 5.**
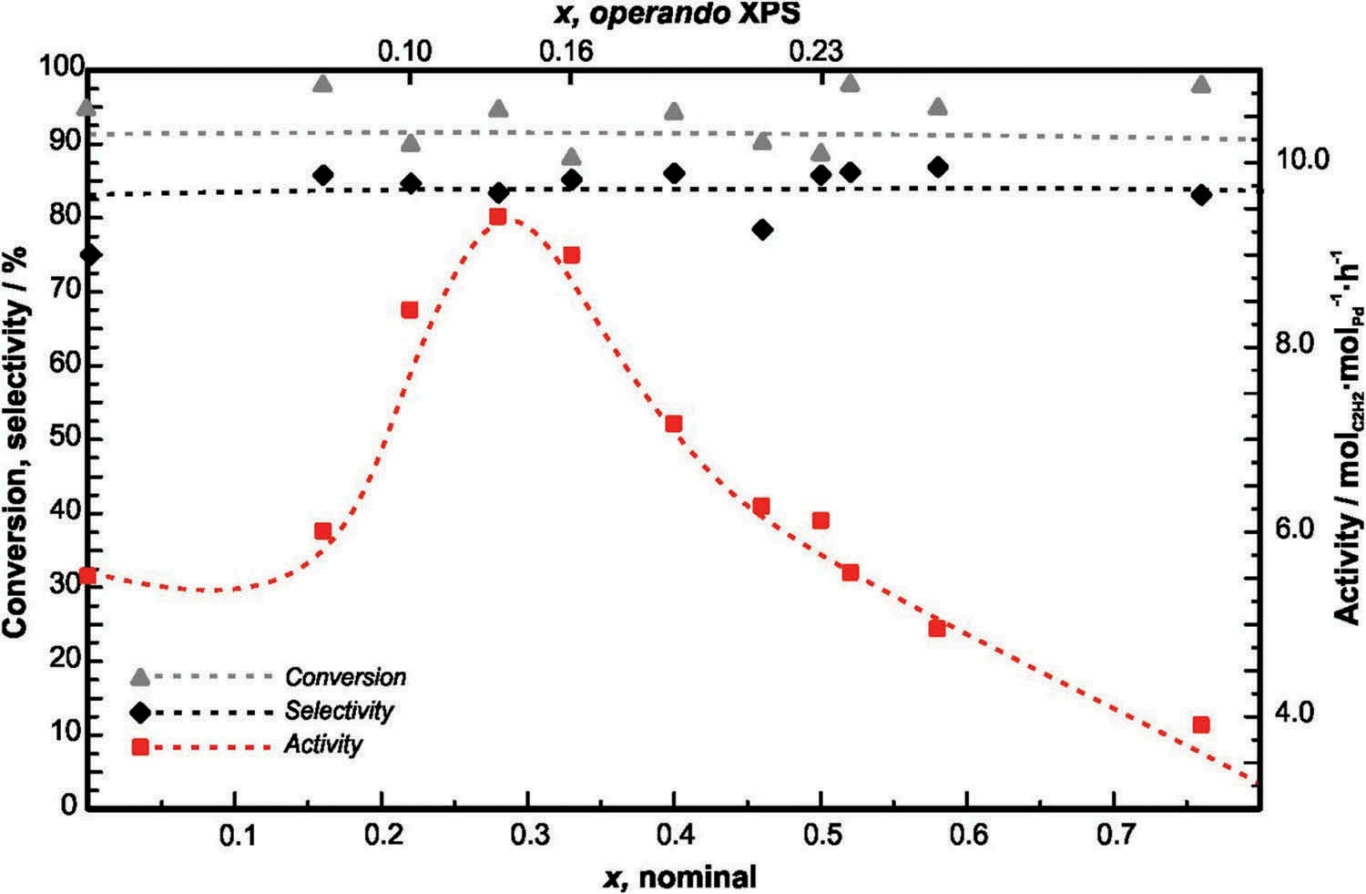
Catalytic properties of Ga_1-*x*_Sn*_x_*Pd_2_ in the semi-hydrogenation of acetylene. Conversion, selectivity to ethylene and activity of the samples are shown over the nominal composition and the composition of the near-surface region under reaction conditions as determined by XPS. Dashed lines are a guide to the eye. Reprinted with permission from [35]. Copyright 2018 American Chemical Society.

As expected, the compounds revealed an excellent ethylene selectivity. However, the highest selectivity was not observed for the ‘fully isolated’ GaPd, but for GaPd_2_ offering in addition 100 times higher activity than GaPd [30]. The small differences in selectivity suggested a minor influence of the electronic structure, which was addressed after a full isostructural series of (Ga,Sn)Pd_2_ could be established[35]. The partial substitution of gallium by tin results in a higher valence electron concentration, thus changing the filling degree of the electronic structure scaffold in very small steps. The segregation behaviour of the different members of the series was established by *operando* X-ray photoelectron spectroscopy (XPS) thus enabling the quantitative correlation of the activity and selectivity to the electronic structure (Figure 5).

**Figure 6.**
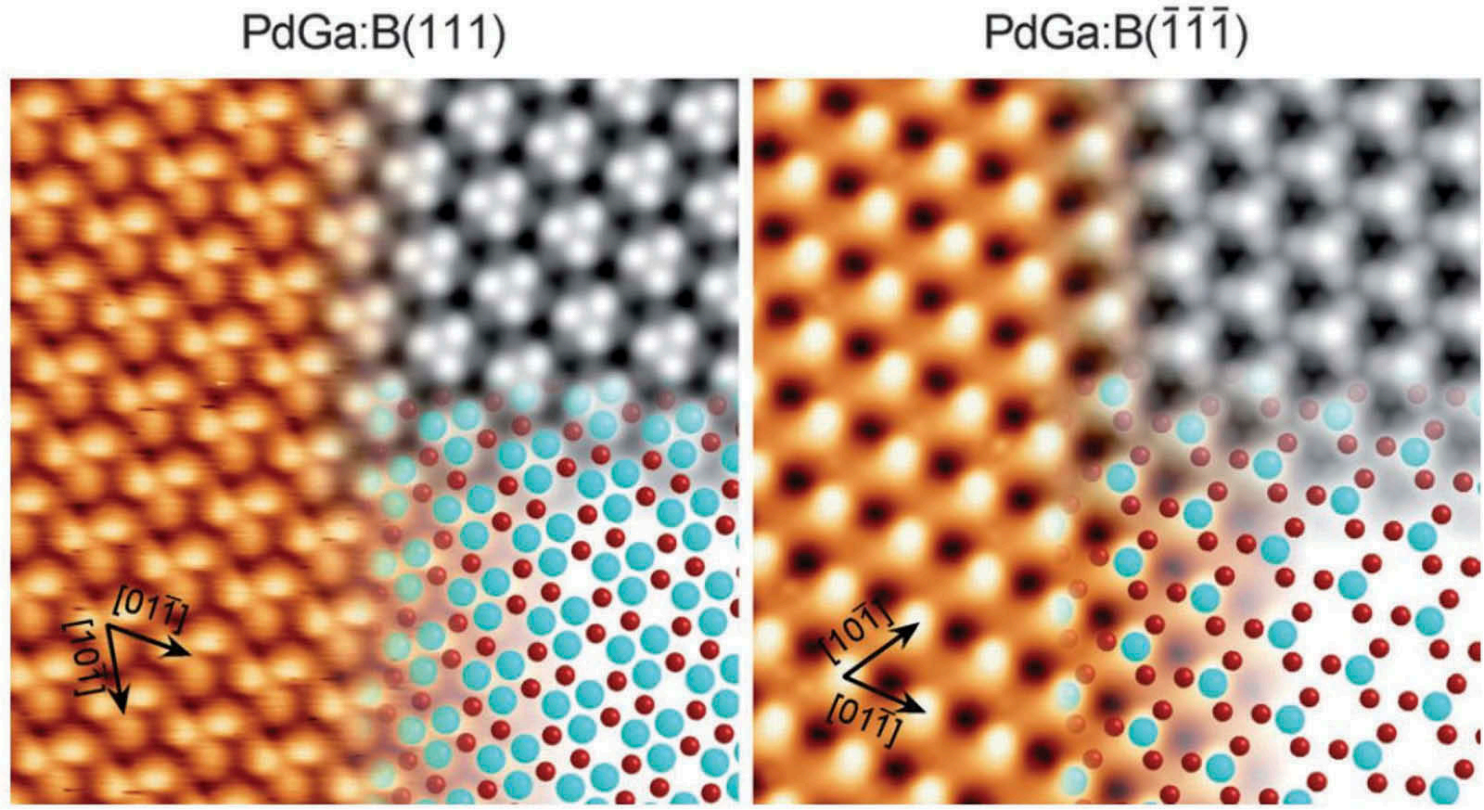
Experimental scanning tunneling microscopy images (*T* = 77 K) overlaid with simulated images (top right inset) and the respective structure models (bottom right inset; Pd = aqua, Ga = red) for PdGa:B(111)Pd3 and PdGa:B($\overset{ˉ}{1}\overset{ˉ}{1}\overset{ˉ}{1}$)Pd1. For details on the surface notation see [65]. Reprinted with permission from [65]. Copyright 2012 John Wiley and Sons.

The gained knowledge allowed further developments, e.g. identification of the intermetallic compounds Al_13_Fe_4_ and Al_13_Co_4_ as noble-metal free hydrogenation catalyst[59]. Driven by the availability of single crystals of many materials [60–62], further understanding is established in semi-hydrogenation reactions by surface science studies [63–67]. With the aid of atomically resolved scanning tunnelling microscopy, the surface structure can be revealed (Figure 6) which then forms the basis for reliable quantum chemical calculations [68–71]. While covalent bonding in intermetallic compounds can stabilise bulk-terminated surface structures this is no necessity. Development in the synthesis of nanostructured materials enabled high-performance materials [43,72], and the deep understanding uncovering the hydrogenation properties of BaGa_2_, the first intermetallic hydrogenation catalyst not containing transition metals[73]. Common to all the different materials forms is their identical selectivity, resulting from the identical underlying crystal structure [74,75], proving the applicability of intermetallic compounds as platform materials under strongly reducing conditions.

Leaving these strongly reducing conditions, intermetallic compounds are more prone to adapt to the chemical potential of oxygen, nitrogen or other elements in the gas-phase by (reversible) partial or full oxidation. While partial oxidation results in the presence of two or more phases (intermetallic compound and oxide/hydroxide/nitride/ …) which can contribute to the catalytic properties, exploiting the latter leads to high-performance materials.

**Figure 7.**
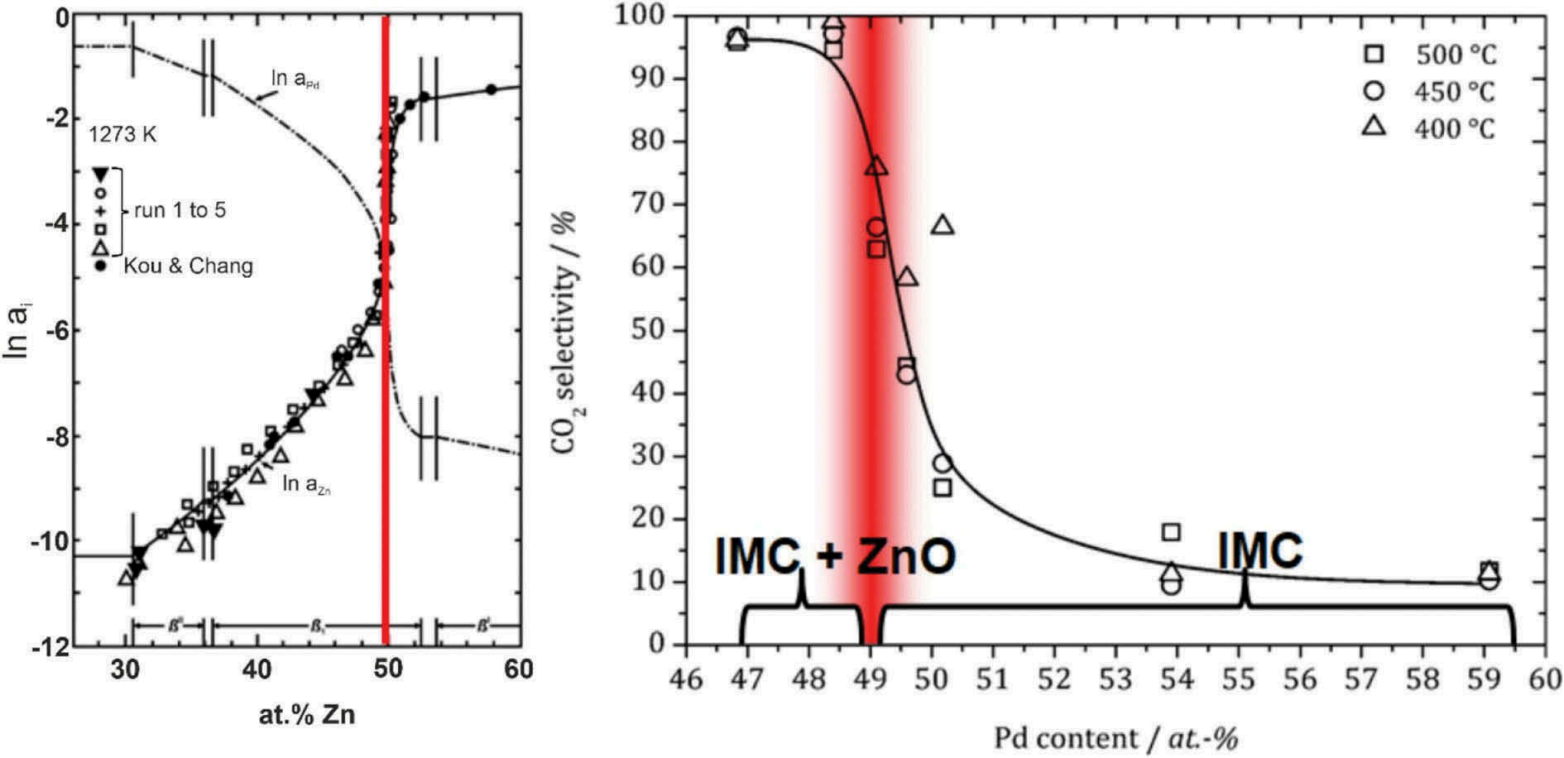
Chemical activity of zinc in the Pd-Zn system (left, modified from [82]) and CO_2_ selectivity in dependence of composition and detected ZnO by *operando* XPS (right, modified from [80], copyright 2012, with permission from Elsevier) A clear correlation between the chemical activity and the catalytic properties is seen.

Methanol steam reforming (CH_3_OH + H_2_O → CO_2_ + 3 CO_2_) is a reaction central to the methanol economy [76,77] and the needs of the reaction have been addressed in detail using intermetallic compounds[2]. As shown in the case of ZnPd/ZnO, an intimate mixture of a (inter)metallic (likely responsible for C-H activation and H-H formation) and an oxidised species (activating H_2_O) is needed to achieve high activity and selectivity[78]. The metallic species is decisive for methanol being activated towards methanol decomposition (resulting in high CO-selectivity) or methanol steam reforming. While elemental palladium leads to decomposition of methanol, the altered electronic structure of ZnPd leads to high CO_2_-selectivity – if ZnO (possessing a high CO_2_-selectivity [79]) is in close proximity[80]. ZnPd possesses a broad homogeneity range, i.e. it can be obtained as single-phase material keeping the CuAl type of crystal structure at different Pd:Zn ratios[81]. The different composition results in different chemical potential of the two elements, which changes strongly at the 1:1 ratio (Figure 7) [82,83].

For zinc-rich compositions of more than 51 at-% zinc, the chemical potential concerning oxygen in the feed is sufficient to partially oxidise the compound on the surface, resulting in the presence of Pd-enriched ZnPd and ZnO. The Pd-enriched ZnPd can not be further oxidised, thus results a mixture of ZnPd and ZnO leading to high CO_2_-selectivity in the reaction (Figure 7). For compositions with more than 49 at.-% of palladium, the chemical potential of zinc in the intermetallic compound changes such that the zinc resists oxidation by the reaction mixture. This results in a lack of the oxidised species, thus water cannot be activated and only methanol decomposition (CH_3_OH → CO + 2 H_2_) takes place on the catalyst´s surface leading to very low CO_2_-selectivity. Drawback of the Pd-Zn system is the quick adaption to reaction conditions requiring *operando* investigations to prove the phases present under reaction conditions. A recent development is to use intermetallic Cu-Zr samples to gain further understanding of the influence of the metal-oxide interface [84,85]. Here, the material dynamics under reaction conditions are expected to be ‘one-way’ due to the large thermodynamic barrier for the reduction of Cu/ZrO_2_ formed under *operando* conditions.

An exciting new development within using intermetallic compounds to gain deep insight into catalysis is testing Heusler compounds for their catalytic properties. Heusler compounds are typically ternary *X*_2_*YZ* intermetallic compounds, where *X* is a group 8–12 transition metal, *Y* a group 3–8 transition metal and *Z* is a main group metal from groups 13–15. The structural scaffold of the class of Heusler compounds allows for extensive substitution of the constituting elements, thus enabling to test for electronic influences in different reactions. Up to now, this approach has been applied to the magnetic properties as well as in the selective hydrogenation and methanol steam reforming. Especially in the latter, the stability of the compounds seems to be an issue. A recent review on this topic has been published by Kojima[86].

### 3.2. High-performance materials

Owing to the great success of intermetallic catalysts many groups are working on the synthesis of high-performance materials[87]. Here, one challenge is the controlled synthesis of supported nanoparticles of the intermetallic compounds or their deliberate decomposition to enhance atom efficiency, thus involving cases 1 to 3 (Figure 3). While the developments in synthesis are focused on in the next section, developments in catalysis are highlighted here.

The electrocatalytic application of intermetallic compounds started already back in the 1970s [88]. However, new concepts and design principles for nanoparticulate materials, i.e. shape- and size control, led to new opportunities for the application of these materials[43], especially in the field of energy relevant electrocatalysis [4], the latter being driven mainly by the prospering field of low temperature fuel cells [4,89,90]. After the early patent by Siemens AG [88], claiming PtPb (NiAs-type) as catalyst material for several electrocatalytic reactions, the Pb-Pt system was further investigated by several groups [91–94]. Sun et al. [95] . enhanced the activity even more by engineering defects and partially amorphous regions into otherwise ordered PtBi nanoplates. The degree of disruption of the ordered structure is achieved by sputtering with different C^+^ ion fluxes. The authors demonstrate the interface between crystalline and amorphous domains, as well as defects and domain boundaries within the crystalline structure, to be responsible for mass activity enhancements by factors of 1.91, 1.82 and 3.0 over the pristine PtPb nanoplates in the methanol oxidation reaction (MOR), ethanol oxidation reaction (EOR) and the oxygen reduction reaction (ORR) under acidic conditions, respectively.

**Figure 8.**
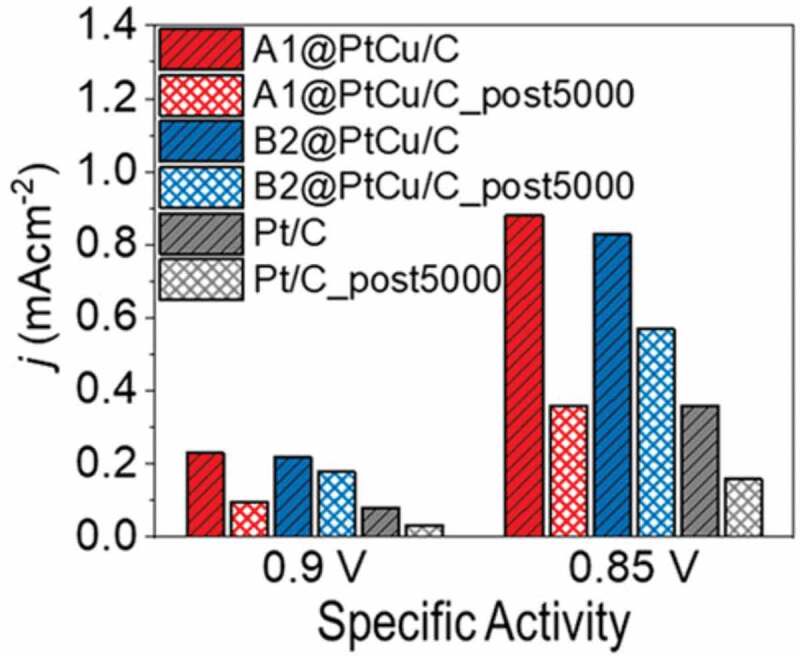
Specific activities at 0.85 and 0.9 V before and after ADT of disordered (A1) an ordered (B2) PdCu cores coated with a PtCu shell. Reprinted with permission from [97]. Copyright 2019 American Chemical Society.

Especially in reactions like the ORR, where a comparably high potential of up to 1.1 V vs. reversible hydrogen electrode (RHE) is necessary, catalytic materials are prone to dissolution under operational conditions. In most cases, it can be stated that ordered crystal structures are more favourable over their disordered counterparts in terms of activity and stability, usually also resulting in a higher durability. Another common concept, applicable independent of the crystal structure, is the formation of Pt shells around a less platinum containing core – a strategy developed by Strasser [96]. A recent contribution by Gamler et al. [97] . combined both concepts by using ordered face-centred tetragonal PdCu (CsCl-type) and disordered face-centred cubic PdCu (fcc, Cu-type) seeds as cores for disordered fcc-Pt-Cu (Cu-type) shells. By isolating the crystal structure of the cores to be the only difference between both model systems, a significant influence of the ordered crystal structure could be revealed, resulting in more stable particles having an intermetallic core after 5000 cycles of an accelerated durability test (ADT) by means of specific activity (Figure 8) but also in terms of copper leaching. The authors ascribe the higher durability to a strengthened interaction between ordered core and disordered Pt rich shell, which is supported by molecular dynamics simulations.

Pt/Pd-*M* based ORR catalysts are usually used in the ratios of 3:1 and 1:1. The former being more stable, as it is more likely to form a noble metal shell around the intermetallic compound core, and the latter having a higher initial activity [98,99]. Sun et al. [100] . exploited the instability of a mixture of α- and β- PdBi_2_ (PdBi_2_ and CuZr_2_ type) nanoparticulate precursors for an *in situ* transformation to obtain Pd_3_Bi (Pd_3_Bi-type). The latter shows superior ORR activity over Pd/C and Pt/C in alkaline media and is an ideal candidate as cathode in the direct methanol fuel cell (DMFC), as it is inactive in the electrochemical oxidation of methanol. Methanol crossover in DMFCs and the resulting short circuit reaction on the cathode is one of the challenges to overcome in DMFC development.

**Figure 9.**
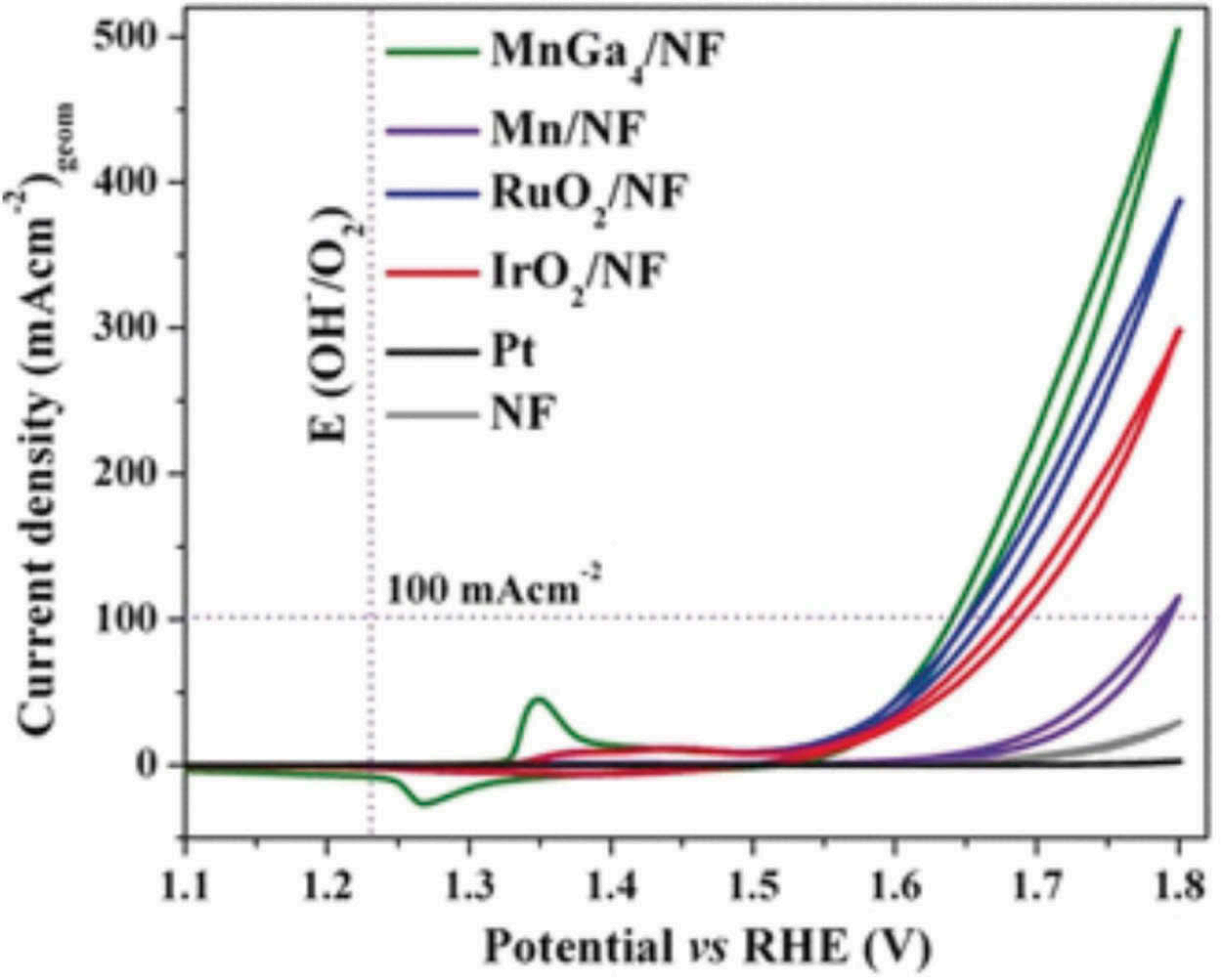
OER catalytic performances of intermetallic MnGa_4_, metallic Mn, and bare nickel foam (NF). Reproduced from [101] under creative commons CC BY 4.0 license.

The oxidation of water (oxygen evolution reaction; OER), usually catalysed by noble transition-metal oxides and applied potentials above 1.23 V vs RHE, is outside the scope of intermetallic compound catalysts from a stability point of view. However, Menezes et al. [101] . recently demonstrated that the intermetallic compound MnGa_4_ is a superior precursor, which *in situ* decomposes to three different MnO_x_ types obtaining polyvalent manganese species. The abundance of the latter and a high electrochemical surface area leads to superior activity of 10 mAcm^−2^ and 100 mAcm^−2^ at overpotentials of 291 and 402 mV (Figure 9). The precursor MnGa_4_ thus not only outperforms MnO*_x_* prepared by alternative synthesis routes but also state of the art RuO_2_ and IrO_2_.

Many reports on the use of nanoparticulate supported intermetallic materials in gas-phase reactions are available which are covered to some extent in recent reviews [43,89,90]. As in electrocatalysis, leaching of unsupported single-phase intermetallic compounds (not alloys as Raney) has been applied to use them as defined precursors for catalytically active materials. Tsai [102] leached a well-characterized and single-phase Al-Cu-Fe quasicrystalline material with NaOH leading to dissolution of the aluminium. This leaves behind well-dispersed Cu-particles, which possess excellent catalytic properties in the steam reforming of methanol (CH_3_OH + H_2_O → CO_2_ + 3 H_2_). The remaining iron stabilizes the particles against sintering. Important to note is that here the catalytic properties are not connected to the intermetallic compound, but to the leaching products, i.e. elemental copper supported on the insoluble residues after leaching and an intermetallic core, as revealed by the characterisation of the material after leaching and catalysis.

Instead of leaching or synthesis of supported intermetallic compounds to gain finely-dispersed active material, bulk intermetallic compounds can also be used as precursors to be deliberately decomposed under reaction conditions. Subjecting intermetallic Cu-Zr compounds, e.g. Cu_51_Zr_14_ to methanol steam reforming conditions results in decomposition[103]. Upon contact with the methanol/water mixture, the intermetallic compounds are partially oxidised, resulting in elemental copper nanoparticles supported on ZrO_2_, which represents a highly active and CO_2_-selective material (>99%). While the conversion of the methanol/water mixture results in a hydrogen-rich product stream (CO_2_:H_2_ = 1:3), the reduction potential is too low to reduce the ZrO_2_ back to intermetallic compounds. At the same time, the copper particles are not oxidised and the high selectivity can be ascribed to the large concentration of interface between the copper particles and the ZrO_2_. Other approaches to use unsupported intermetallic compounds as precursors utilise the hydride formation ability of many intermetallic compounds. Due to the hydride formation, the hydrogen molecules are activated by splitting the H-H bond. These materials, which often contain rare-earth metals, e.g. LaCu_5_ or LaNi_5_, have been described to hydrogenate elemental nitrogen to ammonia [104] or H_2_/CO mixtures to methanol [105]. While in the first case, the intermetallic compound or hydride is not decomposed, the reaction conditions in the latter case typically result in transition metal nanoparticles supported on the corresponding rare-earth oxide, e.g. Cu/La_2_O_3_[106]. In this case, the catalytic properties are not intrinsic to the intermetallic compound or the hydride, but to the supported transition metal and the oxide. The specifics of intermetallic rare-earth compounds in catalysis and the involvement of the corresponding hydrides, nitrides and oxides in the methanol and ammonia synthesis, methanation, Fischer-Tropsch catalysis, (de)hydrogenations as well as electrocatalysis were reviewed recently[107].

Depending upon the conditions in the gas-phase, intermetallic nanoparticles will not decompose and can be used with their specific properties for catalytic conversions. To exploit the specific catalytic properties, the single-phase synthesis of the intermetallic nanoparticles is required and poses a major challenge (see section ‘Synthesis’). This holds for intermetallic compounds which are formed under reaction conditions (see next section) and also for those that are stable as bulk compounds under reaction conditions. For ZnPd [108] in methanol steam reforming as well as GaPd_2_ [72] and GaPd [109] for the selective hydrogenation of acetylene, highly active materials have been developed based upon a targeted nanoparticulate synthesis. Common to both is that the high-performance materials keep the excellent selectivities while the activity per Pd-atom is boosted by as much as a factor 32.000.

Intermetallic Ga-Ni [110,111], Ga-Pd [112] and Zn-Pd [113] compounds were applied successfully as catalysts to synthesise methanol from CO_2_ and hydrogen at normal pressure – thus opening a condition window which enables to store the fluctuating electricity supply from renewable sources like windpower and photovoltaics in chemical form. In this important step of the methanol economy [114], the CO_2_ is ideally sequestered from air and the necessary hydrogen comes from electrochemical water splitting. Characterisation of the materials after reaction reveal the stability of the intermetallic compounds under reaction conditions.

### 3.3. New approaches

**Figure 10.**
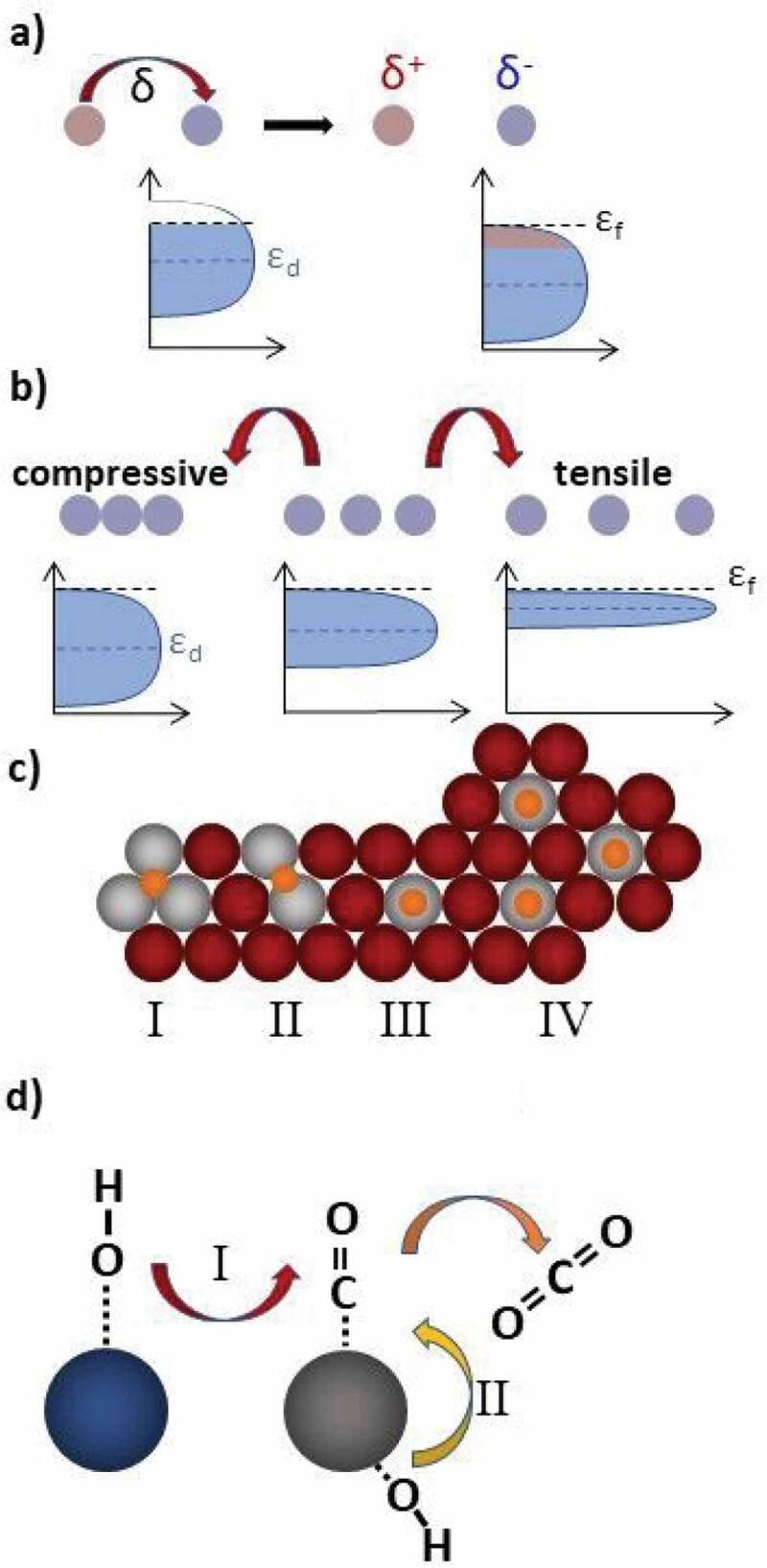
Strategies for catalytic development offered by intermetallic compounds: ligand effect (a), strain effect (b), geometric effect (c) and bifunctional mechanism (d). Geometric effects (c) are divided into two parts. The first is the active-site isolation effect, accompanied by different adsorption possibilities of a molecule, i.e. in the hollow site (cI), on the bridge site (cII) or on top of an atom (cIII). The second is the equality of sites (cIV). The bifunctional mechanism (d) proceeds via an initial spill-over of species (here OH*, red arrow) to enable teamwork. Reprinted with permission from [4]. Copyright 2019 American Chemical Society.

Intermetallic compounds have been applied to test for electronic and structural influences in electrocatalysis and selective hydrogenations. These approaches exploit the characteristic properties of intermetallic compounds (ligand, strain and geometric effects), while the advantage of adjusting the chemical potential in a wide range has been used to gain understanding in the teamwork between metallic and oxidic species in methanol steam reforming and electrocatalysis (bifunctional mechanism, Figure 10). Of course, these approaches are just first examples or proof of principle and can be extended to other reactions.

Besides these, new approaches to use intermetallic compounds to accomplish materials with specific catalytic properties and to answer open questions are under development. Extending the active-site isolation concept has led to many materials presenting not only homogeneous active sites, but enabling single-atom catalysis on which a recent review is available[115]. The ordered crystal structure of the intermetallic compounds results in a full dispersion of the active metal on the surface of the materials and only one type of environment. A still open question is to which extent the ‘matrix’ of the inactive metal is really inactive. Upon intermetallic compound formation, the nature of the constituting elements changes, thus e.g. hindering intermetallic palladium in the intermetallic Ga-Pd compounds from hydride formation, making it a tamed hydrogenation catalyst. On the other hand, quantum chemical calculations show that it is rather likely that the intermetallic gallium in these compounds is taking an active part in the surface diffusion of atomic hydrogen[116].

Structural influences have been addressed using silica-supported palladium-based catalysts for the hydrogenolysis/isomerization of neopentane and propane. [117] While elemental palladium – presenting large active sites – resulted in high neopentane hydrogenolysis, addition of zinc and the subsequent formation of the intermetallic compound ZnPd diminishes the activity drastically and leads to dehydrogenation. Thus, a direct correlation between the selectivity and the size of the reactive ensemble could be shown. This demonstrates that hydrogenolysis requires a particular reactive ensemble whereas propane dehydrogenation does not. While in this study the electron concentration varied by introducing zinc into the palladium, order-disorder transitions at constant composition can be employed to keep the electron count constant. Several bimetallic systems offer such transitions, among them Ag-In at the 3:1 ratio and Cu-Pd at the 1:1 ratio. Such Ag-In materials (ordered: Cu_3_Au type of crystal structure; disordered: Cu type of crystal structure) have been applied to test for structural influences on the reduction rate of para-nitrophenol to para-aminophenol using NaBH_4_ as reduction agent[118]. The higher the structural order, the slower the reaction proceeds. Furukawa et al. investigated the chemoselectivity in the hydrogenation of nitrostyrene providing polar active sites to enhance the hydrogenation of the polar nitro-group without hydrogenation of the C = C bond[119]. A large number of supported Rh- or Pd-based materials was tested resulting in the identification of RhPb_2_ and Pd_13_Pb_9_ as superior catalysts with a selectivity towards aminostyrene of 91% and 92%, respectively.

**Figure 11.**
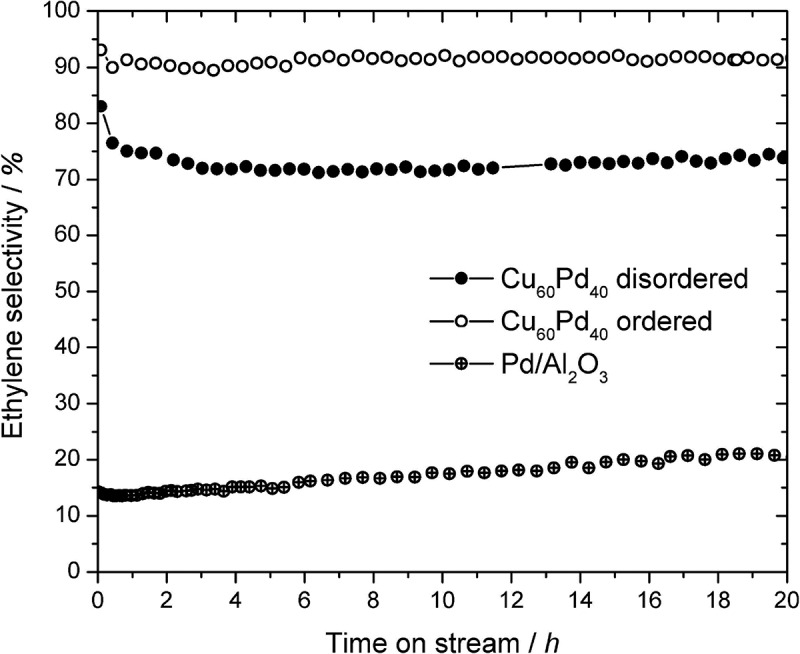
Selectivity to ethylene of ordered and disordered Cu60Pd40 (5 mg each) and 5% Pd/Al2O3 (0.5 mg) in the semi-hydrogenation of acetylene at 200°C (C2H2:H2:C2H4 = 1:10:100). The catalysts were tested without any pretreatments. The experimental error lies within the radii of the depicted symbols. Reproduced from [120] under creative commons CC BY license.

Also, the effect of ordering on the selectivity can be explored. Using Cu_60_Pd_40_ nanoparticles in the disordered (cubic close packed) and ordered form (CsCl type of crystal structure) in the selective hydrogenation of acetylene reveals a 20% higher selectivity of the ordered intermetallic compound in comparison to the disordered alloy (Figure 11)[120].

The interplay between the reaction mixture and the chemical potential of the intermetallic compounds can be very surprising. Recently, a rather complex relation has been revealed using intermetallic Ag-Ca compounds as catalyst precursors in the epoxidation of ethylene (2 C_2_H_4_ + O_2_ → 2 C_2_H_4_O) [121,122]. The study aimed at providing finely dispersed silver particles supported on CaO by partial oxidation of the intermetallic compound by the reaction mixture. Intermetallic compounds in the system (Ca_2_Ag_7_, CaAg_2_, CaAg, Ca_5_Ag_3_ and Ca_3_Ag) were synthesised, tested and characterised before, during and after catalytic testing. In contrast to expectations, the higher the calcium content the more stable the materials became under reaction conditions. While this cannot be explained by the chemical potential, quantum chemical calculations showed that the strong adsorption of ethylene on the intermetallic surfaces hinders oxidation, thus stabilising the surface against the reactive atmosphere. As expected, this behaviour is not isotropic and a contribution gives all the details of this interesting interplay[123].

An interesting branch opened very recently in the activation of nitrogen and the subsequent synthesis of ammonia. The discovery of ammonia synthesis catalysts based on the electride Ca_24_Al_28_O_64_ in 2012 by Kitano et al. [124] . inspired studies on the intermetallic compound Y_5_Si_3_ [125]. In both cases, electrons are transferred to nitrogen with the help of supported ruthenium nanoparticles, thus reducing the apparent activation energy, and achieving an activity of 1.9 mmol g^−1^ h^−1^ at 400°C and ambient pressure for the latter. Very recently, ammonia synthesis over LaCoSi – without the help of Ru – was reported and analysis after reaction showed no additional phases, thus allowing to allocate the observed catalytic properties to the intermetallic compound itself[126]. Quantum chemical calculations showed that the reaction proceeds via an electron transfer from the negatively charged Co atom to nitrogen, and the arrangement of the atoms stabilises the nitrogen adsorption, thus reducing the apparent activation energy and resulting in an activity of 1.25 mmol g^−1^ h^−1^ at 400°C and ambient pressure. Besides the developments listed here, many more might currently be in progress in this very vivid field of heterogeneous catalysis (Figure 2).

## 4. Developments in synthesis

Especially for catalysis, it is advantageous that intermetallic compounds can be synthesized on different length scales, covering nanoparticles, polycrystalline powders as well as large single crystals. Since intermetallic compounds possess specific crystal structures with a high preferential site occupancy (due to the charge transfer and chemical bonding), the different forms result in very similar chemical properties. This allows to use the compounds as platform materials, since the easy-to-obtain and straightforward-to-characterise unsupported polycrystalline powders allow to determine the intrinsic catalytic properties, while surface science studies on single crystals in combination with quantum chemical calculations enable understanding of the ongoing processes and supported nanoparticles result in high-performance materials which can be applied in industrial processes. This section starts with the synthesis of polycrystalline bulk materials, moving then to (thin) films and large single crystals before summarising synthesis routes to (un)supported nanoparticles.

### 4.1. Bulk materials

Synthesis of bulk polycrystalline materials is in most cases straightforward and results in materials which can be well characterised before and after catalytic tests. The presence of only one phase also enables *operando* characterisation, i.e. following any changes while the catalysis is running. Basis for the synthesis are the corresponding phase diagrams and bulk intermetallic compounds are usually obtained by high-temperature methods. This comprises melt synthesis (e.g. arc melting) or powder metallurgical methods, i.e. annealing for appropriate time in inert crucibles and atmosphere, to reach thermodynamic equilibrium. The resulting bulk materials are very often brittle, and by crushing or milling a specific surface area in the order of 0.01 to 0.1 m^2^ g^−1^ can be achieved. Such materials are ideally suited for basic studies to understand ongoing processes as well as establishing a benchmark concerning the intrinsic catalytic properties.

**Figure 12.**
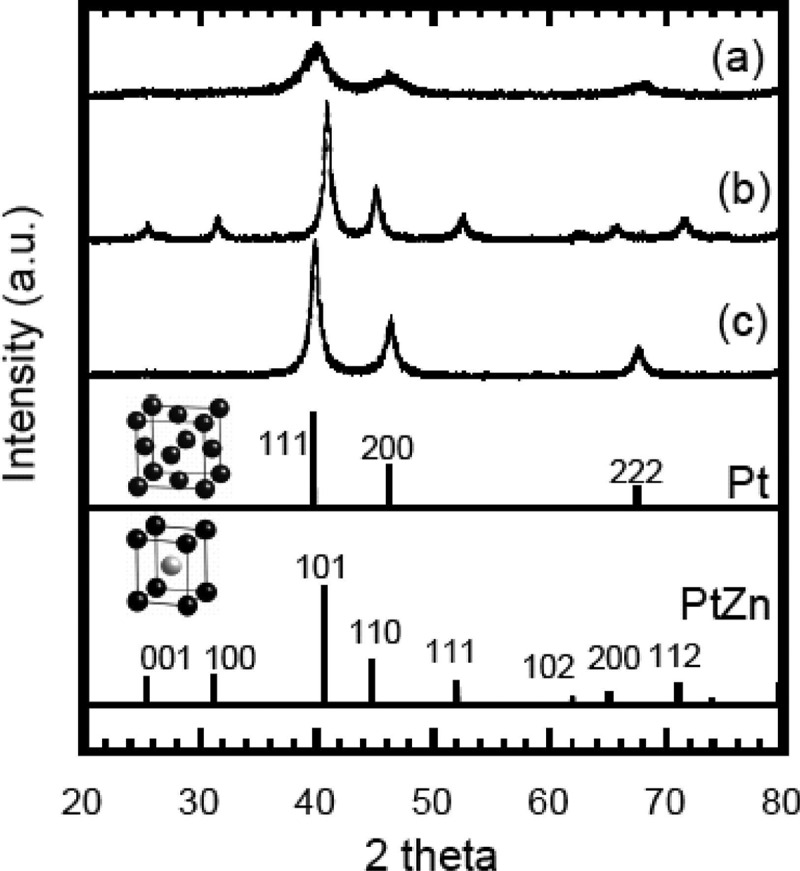
Powder X-ray diffraction (pXRD) pattern of (a) unheated Pt/C (Vulcan), (b) PtZn/Vulcan synthesized by heating Pt/Vulcan with a Zn chip, (c) Pt/Vulcan heated without a Zn chip. Pt (JCPDS: 04-001-0112) and PtZn (JCPDS: 01-072-3027) are shown. Reprinted with permission from [133]. Copyright 2009 American Chemical Society.

Bulk materials can also often be – and sometimes have to be – synthesized by non-conventional methods. Reasons might be sluggish diffusion kinetics, high vapour pressure differences or very high melting temperatures. Such non-conventional synthesis methods comprise chemical vapour transport reactions [127] (allowing also for the synthesis of mm-sized single crystals), vapour-solid synthesis [128] (enabling the synthesis of bulk foils, e.g. for electrocatalysis) with the isopiestic method being a special case of the latter one [129]. By chemical vapour transport reactions, a large variety of intermetallic compounds has been obtained. A recent development is the possibility to activate boron, thus opening new synthesis routes with this element[130]. The only drawback using this synthesis method might be the presence of traces of transport agent on the surface, which can alter the adsorption properties. The high vapour pressure of many metals (In, Zn, Hg, Cd or Sb just to name a few) is a challenge in the bulk synthesis of intermetallic compounds containing such metals. Typically, some amount of these metals is lost, resulting in samples which are deviating from the aimed-for composition and containing two phases in the worst case. The vapour-solid synthesis turns this drawback into an asset. The two metals are placed separated in a closed vessel and then heated to a temperature where the vapour pressure becomes significant. Reaction of the vapour with the second metal (powder, foil or granules) sets in, lowering the vapour pressure and thus enhancing the evaporation of the high-vapour-pressure metal until all has reacted with the second metal. Subsequently, the temperature can be increased to accelerate the diffusion in the solid sample and to reach thermodynamic equilibrium. The method has been successfully applied to the synthesis of foils of intermetallic compounds in the systems Pd-Zn [131], Cu-Zn [128] and Pt-Zn [132] for electrochemical investigations. These can not be obtained by pressing or cold rolling from ingots due to the high brittleness of the intermetallic compounds. Providing the second metal as supported nanoparticles, the vapour-solid method can also be applied to obtain supported intermetallic nanoparticles (Figure 12)[133].

If thin films of intermetallic compounds are required, either magnetron sputtering can be applied [134,135] or so-called near-surface intermetallic phases can be synthesised[136]. In principal, magnetron sputtering should result in quick and reproducible synthesis of thin intermetallic films by co-deposition or subsequent sputtering and subsequent annealing to reach thermodynamic equilibrium. While this approach works for some intermetallic compounds (e.g. Pt_2_Si [134]), others are not formed or deposition of films of several hundred micrometre thickness are required (e.g. GaPd_2_ [137]). In some of these cases, the use of an intermetallic target instead of elemental targets can help[135], while there are also known cases, where even this does not work (e.g. Al_13_Fe_4_). The underlying reasons why sometimes sputtering is straightforward and sometimes not are not understood yet. By deposition of wedges, sputtering also allows synthesis of thin films with concentration gradients. This results in libraries of binary or ternary systems, which can then be catalytically tested (e.g. Bi-Pb-Pt for direct methanol fuel cell anodes [138]). In all cases, characterisation of the thin films is a challenge and requires methods which are sensitive to the near-surface region. Surface-science studies can be conducted on so-called near-surface intermetallic phases (NSIPs)[139]. An NSIP is obtained by deposition of a small number of monolayers of a second metal onto a single-crystalline substrate in ultra-high vacuum and subsequent annealing so that the two metals can react in the near-surface region, i.e. a couple of atomic layers. Products can be alloys or ordering occurs and an NSIP is formed. While these can be characterised thoroughly by surface-science methods also concerning their catalytic properties, one has to keep in mind that the electronic structure is influenced by the underlying metal and that the NSIPs are thermodynamically not stable. Extensive heating will either lead to evaporation of the second metal or it diffuses into the bulk of the single crystal. Examples for studies on NSIPs are Ga-Pd [139], Pd-Zn [136] or Pt-Zn [140].

### 4.2. Large single crystals

To study surface properties by surface-science methods without the influence of an underlying substrate, large single crystals of intermetallic compounds are required. A large number of compounds has been grown as cm-sized single crystals over the years. Techniques for growing large intermetallic single-crystals involve growth by the Czochralski (e.g. in the Al-Cr-Fe system [141]) or Bridgeman (e.g. in the Yb*TX* system with *T* = Cu, Ag, Pt or Au and *X* = Sn or Sb) techniques as well as synthesis from metallic fluxes. Synthesis from metallic fluxes involves the slow cooling of a suitable melt to obtain large crystals[142]. The last step is to isolate the crystals from the (solidified) melt. This is usually done by dissolution of the melt by suitable acids or bases with the risk that the intermetallic compounds are also dissolved. Instead, the single crystals can be obtained from the liquid melt by ‘sieving’ the sample at high temperature in a centrifuge[143]. For samples grown from tin melts, isolation of the crystals is possible employing the β- to α- tin transition below 13°C which leads to a large volume expansion upon which the metallic matrix is crumbling away leaving clean single crystals behind[28].

### 4.3. Supported nanoparticles

The strong increase in the interest of the catalytic properties of intermetallic compounds has caused a large boost in the number of synthesis routes available to supported intermetallic nanoparticles. The challenge here is to develop synthesis protocols which result in only one intermetallic compound and to proof this by thorough characterisation of the materials (see also next section). If more than one phase is present, assigning the catalytic properties to one compound becomes impossible – especially if elemental noble metals contribute to the catalytic properties.

To form intermetallic nanoparticles, at least two metals are necessary – besides the thermodynamic requirements, i.e. the presence of an ordered crystals structure at the ratio of the two metals. During synthesis, both metals can be supplied at the same time or in subsequent steps, where the latter synthesis protocols usually result in better defined materials. Supplying both metals at the same time can be done by co-impregnation of the support followed by reduction with the risk that the metals end up in different ratios in the formed particles or that the harder-to-reduce metal is not fully available in the metallic state, thus altering the real composition from the nominal one. Co-impregnation does usually not result in single-phase intermetallic particles and only in few cases the synthesis protocols can be developed to a state where supported single-phase materials are obtained (e.g. FePt [144]).

**Figure 13.**
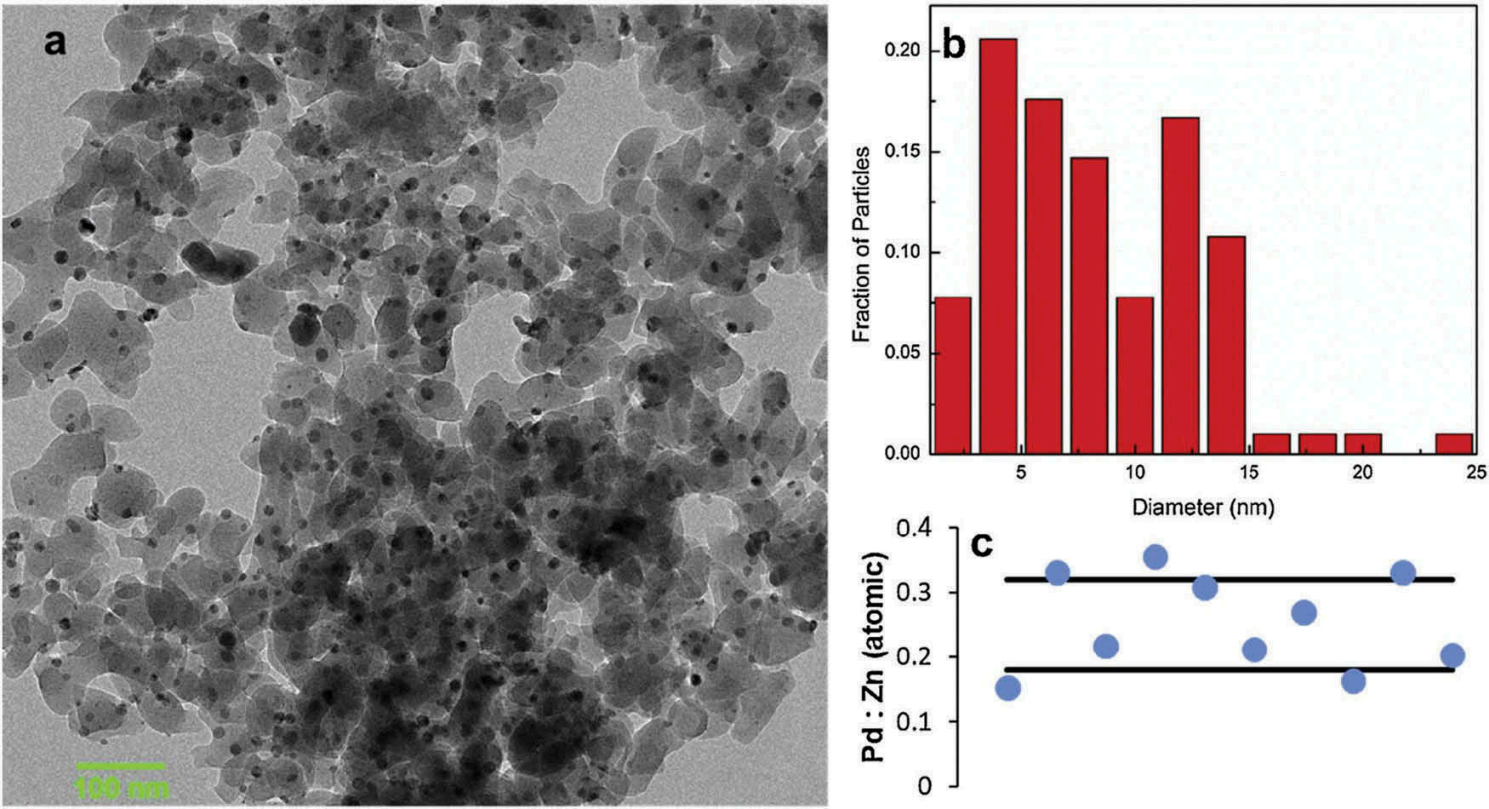
(a) TEM image, (b) particle size distribution and (c) individual particle-by-particle composition of γ-brass Pd-Zn/SiO_2_, determined by scanning transmission electron microscopy combined with energy-dispersive spectroscopy (STEM-EDS) using the parent 5% Pd/SiO_2_. Black lines in (c) indicate the compositional bounds of the Pd-Zn γ-brass phase. Reprinted from [145], copyright 2019, with permission from Elsevier.

More versatile and reliable are routes in which the metals are subsequently introduced. Starting point is a support on which metal A is present in nanoparticulate form. Subsequently, metal B is introduced in such a way that it is only present at the nanoparticles of A and at the same A:B ratio. While this is possible by a second impregnation, the challenges are the same as with the co-impregnation mentioned above. Metal B can also be introduced by the vapour-solid method (see section ‘Bulk Materials’). Reaction times are much shorter than for bulk materials and the feasibility of this approach has been shown for transforming Pt/C to ZnPt/C[133]. The composition of the resulting particles is fixed by the amount of metal B provided in the closed system, thus resulting in excellent compositional control as also shown for silica supported γ-brass phases in the systems Pd-Zn, Cu-Zn and Ni-Zn (Figure 13)[145].

Besides this synthesis route, intermetallic compounds in a supported state can also be obtained by chemical vapour transport reactions. The reaction takes place in a closed vessel and the decomposition of the transport species of metal B at the supported metal A nanoparticles ensures the direct reaction between the two metals, thus leading to the sought-for single-phase intermetallic materials, e.g. GaPd_2_/C [146]. Decomposition of a metal B precursor at the metal A nanoparticles is also used in the chemical vapour and atomic layer deposition (CVD and ALD, respectively). In contrast to the chemical vapour transport reactions, the transporting species is not re-formed in CVD or ALD after delivery of metal B to metal A. CVD is broadly applicable and a few examples are given here: Chemical vapour deposition has been applied to obtain PdFe/SiO_2_ from reacting ferrocene with Pd/SiO_2_ at 90°C and subsequent annealing at 600°C in H_2_ for 1 h[147]. Ni_3_Ge in MCM-41 could be obtained by CVD of Ge(CH_3_)_4_ onto Ni@MCM-41 at 0°C and subsequent annealing at 600°C in H_2_ for 1 h[148]. Several supported compounds in the Ni-Sn system were synthesised by subjecting Ni/SiO_2_ to Sn(CH_3_)_4_[149], and NiSi_2_/SiO_2_ and NiSi/SiO_2_ were obtained by reaction between Ni/SiO_2_ and SiH_4_ [150]. In difference to CVD, ALD is a self-limiting reaction [151]. The selection of suitable reagents and processes makes ALD much more demanding than CVD and has been introduced for the synthesis of intermetallic compounds in 2016 for NiFe films [152]. Since then, it has been applied to synthesise PtSn/SiO2 [153], nanoparticles of PtIn [154] and PtSn [155] as well as thin films of Co_3_Sn_2_ and Ni_3_Sn_2_ [156].

A versatile and elegant method to fulfil the synthesis conditions, is using the reactive metal-support interaction (RMSI) by which many supported intermetallic materials can be obtained. By a partial reduction of the support in the vicinity of the supported nanoparticles of a metal A, metal B atoms are formed which react with metal A to yield intermetallic compounds. The amount of B can be adjusted by the reduction conditions, thus enabling single-phase synthesis of different intermetallic compounds using the same starting material. The process is eased when the reducing agent is activated at the supported metal A (e.g. H_2_ at Pd-nanoparticles) and the support is an easy-to-reduce oxide, e.g. ZnO or In_2_O_3_. But even such refractory oxides as Al_2_O_3_ or ZrO_2_ can be a source of the corresponding metals if the temperature and the reduction potential of the atmosphere are high enough. The method is applicable for many intermetallic compounds and has been reviewed recently[49].

## 5. Characterisation

Ordered crystal structure, as well as its specific physical and chemical properties, is characteristic for intermetallic compounds. The possible change or formation of intermetallic compounds under catalytic reaction conditions requires a thorough characterisation of the material in question at least before and after catalysis in order to learn if the intermetallic compound is changing. Ideally, the stability or instability is corroborated by characterisation while the catalysis is taking place (*operando* characterisation) so that information is obtained under which conditions the intermetallic materials are stable and under which not.

Since the ordered crystal structure is a requirement for an intermetallic compound, the presence can be verified by structure sensitive methods like X-ray diffraction, high-resolution TEM or electron diffraction. In some cases, the intermetallic compound adopts crystal structures, which are very similar to the ones of the constituting elements, e.g. superstructures of close packed structures. In these cases, care has to be taken to detect the (weak) superstructure reflections by choosing appropriate measurement conditions (detection time and angle) in the diffraction experiments or to choose characteristic lattice plane distances in TEM images. Since the combination of the crystal and electronic structure in intermetallic compounds is very peculiar compared to substitutional alloys, it is worthwhile to distinguish between the two material classes properly – it also eases identifying contributions in this vivid field.

Gaining understanding, e.g. by deriving reliable structure-property relationships, requires a structural characterisation of the catalytic materials at least before and after use. To assign catalytic properties to an intermetallic compound requires proving its *operando* stability to exclude decomposition or alteration. This can only be done by *operando* methods (including simultaneous detection of catalytic activity), ideally a combination of bulk and surface sensitive methods, e.g. *operando* XPS and *operando* pXRD. *Operando* measurements are usually laborious and time consuming and often require large research facilities like synchrotrons. Fortunately, suitable stations at synchrotrons have significantly increased in number during the last years, thus easing access to the necessary *operando* methods. Characterisation is important in all cases where intermetallic compounds are (or might be) involved (case 1–3, see Figure 3).

## 6. Conclusions

Intermetallic compounds can be used as platform materials: starting from unsupported, single-phase materials to study the intrinsic catalytic properties, the geometric and ligand effect can be addressed, resulting in optimal materials. These in turn can then be synthesized as supported nanoparticulate materials to study the influence of the support. A large number of publications addresses the catalytic properties of supported intermetallic compounds in well known and new reactions in an explorative way, thus increasing the knowledge of the versatility of this class of compounds.
